# Long non-coding RNA DLGAP1-AS1 promotes the progression of gastric cancer via miR-515-5p/MARK4 axis

**DOI:** 10.1590/1414-431X2020e10062

**Published:** 2021-05-24

**Authors:** Liping Li, Qingjun Lai, Manman Zhang, Jun Jia

**Affiliations:** 1 Department of Medical Oncology, Dongguan People’s Hospital, Dongguan, Guangdong, China

**Keywords:** Gastric cancer, DLGAP1-AS1, miR-515-5p, MARK4, Proliferation

## Abstract

Long non-coding RNA (lncRNA) is an essential regulator of carcinogenesis and cancer progression. In the study, we explored the role of lncRNA DLGAP1-AS1 in gastric cancer (GC). qRT-PCR was carried out to detect DLGAP1-AS1 expression in GC tissues and cell lines. CCK-8 assay, EdU assay, and transwell experiments were employed to detect the malignant biological behaviors of GC cells with DLGAP1-AS1 knockdown or overexpression. Bioinformatics and dual-luciferase report assay were used to confirm the binding relationship between DLGAP1-AS1 and miR-515-5p. MARK4 expression was detected by western blot after DLGAP1-AS1/miR-515-5p was selectively regulated. DLGAP1-AS1 was up-regulated in GC tissues and cell lines, and its high expression was closely associated with larger tumor size, higher TNM stage, and lymph node metastasis. Furthermore, DLGAP1-AS1 overexpression enhanced cell proliferation, migration, and invasion, and miR-515-5p could reverse these effects. DLGAP1-AS1 participated in the regulation of the MARK4 signaling pathway by targeting miR-515-5p. DLGAP1-AS1 promoted GC progression through miR-515-5p/MARK4 signaling pathway.

## Introduction

Gastric cancer (GC) is one of the most aggressive malignancies and the second leading cause of cancer death (1). Although surgical resection and chemotherapy have made some progress in treating GC, relapse and metastasis are still serious prognostic adverse events and the 5-year overall survival rate is still frustrating (2). Elucidating the regulatory network related to the GC progression is of great significance for identifying new biomarkers and developing effective targeted therapies.

Long non-coding RNA (lncRNA) is an RNA transcript that is more than 200 nucleotides in length and cannot encode proteins (3). In recent years, accumulating evidence demonstrates that lncRNAs exert a vital regulatory effect in a variety of diseases, including GC (4). For example, lncRNA FTX is dysregulated in GC, and FTX promotes cancer cell proliferation, migration, and invasion (5). lncRNA TUG1 expression is up-regulated in GC tissues and related to the clinicopathological characteristics (6). It was found that lncRNA DLGAP1 antisense RNA 1 (DLGAP1-AS1) is involved in the tumorigenesis and metastasis of hepatocellular carcinoma (7). Nonetheless, the function and molecular mechanism of DLGAP1-AS1 in GC have not been fully clarified so far.

Microtubule affinity regulating kinase 4 (MARK4) belongs to the microtubule affinity-regulating kinase family, and it phosphorylates microtubule-associated proteins and regulates the transition between stable and dynamic microtubules (8, 9). It is associated with the centrosome throughout mitosis and participates in cell cycle control, so its abnormally high expression contributes to the malignant biological behaviors of cancer cells (8–10). It is reported that microRNA (miR)-515-5p regulates MARK4 in cancer cells, and miR-515-5p/MARK4 axis is implicated in regulating cancer cell migration and metastasis (11). Nevertheless, the role of miR-515-5p/MARK4 axis in GC development has not been reported.

In this research, we explored the role of lncRNA DLGAP1-AS1 in GC.

## Material and Methods

### Sample collection

Our study was approved by the Ethics Review Board of Dongguan People's Hospital. Thirty-one cases of fresh GC tissues and matched normal gastric tissues were collected from the patients who received surgery in Dongguan People's Hospital. The GC patients enrolled in this study received neither chemotherapy nor radiotherapy before the surgery. During surgery, immediately after removal from the patient, the tissues were frozen in liquid nitrogen and stored at -196°C for further analysis. All cases were histologically diagnosed by two experienced pathologists. We obtained written informed consent from all patients before the study.

### Cell lines and culture

Five human GC cell lines (BGC-823, AGS, BGC-803, SGC-7901, and HGC-27), human normal gastric mucosal epithelial cell line (GES-1), and human embryonic kidney cell line (HEK293T) were obtained from the Type Culture Collection of Chinese Academy of Sciences (China). Cells were cultured in RPMI-1640 medium (Thermo Fisher Scientific, China) containing 10% fetal bovine serum (FBS) (HyClone, USA). All cell lines were maintained at 37°C in 5% CO_2_.

### Cell transfection

Negative control microRNA (miR-NC), miR-515-5p mimic, control siRNA (si-NC), DLGAP1-AS1 siRNA (si-DLGAP1-AS1), MARK4 siRNA (si-MARK4), pcDNA3.1 plasmid, pcDNA3.1-DLGAP1-AS1 (pc-DLGAP1-AS1), and pcDNA3.1-MARK4 (pc-MARK4) were available from Genepharma (China). Lipofectamine^TM^ 2000 (Invitrogen, USA) was used to perform transfection according to the manufacturer's instructions. Forty-eight hours after transfection, the cells were harvested for quantitative real-time polymerase chain reaction (qRT-PCR) analysis to evaluate the transfection efficiency.

### qRT-PCR analysis

The total RNA from tissue and cells was extracted using TRIzol reagent (TaKaRa, China) following manufacturer instructions. Total RNA was reversely transcribed into cDNA using a reverse transcription kit (TaKaRa). qRT-PCR was performed with SYBR Premix Ex Taq™ Kit (Takara). To detect the relative expression of DLGAP1-AS1 and MARK4, GAPDH was used as the reference gene, and U6 was used as the reference gene for miR-515-5p. Each sample was processed in triplicate, and the relative expression of genes was calculated using the 2^-ΔΔCT^ method. The primer sequences used in this research are shown in Table 1.

**Table 1:** The primer sequences for qRT-PCR.

Genes	Primer sequences
*DLGAP1-AS1*	
Forward	5′-TTTGATGTTGGCCAATGCCG-3′
Reverse	5′-GGGCAGGGAGTCTTCATAGC-3′
*MARK4*	
Forward	5′-GTCAACAGACTGTGAGAGAGCATCC-3′
Reverse	5′-GCTCTGTGTATGGCTTCAACTCC-3′
*GAPDH*	
Forward	5′-ACCATCTTCCAGGAGCGAGA-3′
Reverse	5′-GACTCCACACCCACTACTCAGC-3′
*MiR-515-5p*	
Forward	5′-TTCTCCAAAAAAAGAAAGCACTTTCTG-3′
Reverse	5′-CTCGCTTCGGCAGCACA-3′
*U6*	
Forward	5′-GCGCGTCGTGAAGCGTTC-3′
Reverse	5′-GTGCAGGGTCCGAGG-3′

### Cell viability analysis

Cell counting kit-8 (CCK-8) (Beyotime, China) was used to determine cell viability. In line with the instructions, GC cells were seeded onto 96-well plates 24 h after transfection (1000 cell/well) and cultured. After 24, 48, 72, and 96 h, 10 μL of CCK-8 reagent was supplemented to each well at each time point, and the absorbance at 450 nm of the cells in each group was measured after 1 h of incubation.

### EdU cell proliferation analysis

Cell proliferation was also assessed using a 5-ethynyl-2-deoxyuridine (EdU) labeling/detection kit (RiboBio, China). GC cells were cultured in 96-well plates (1×10^4^ cells/well). EdU labeling solution was added to the wells, and the cells were incubated at 37°C and 5% CO_2_ for 2 h. After being fixed with 4% paraformaldehyde and treated with 0.5% Triton X-100, the cells were incubated with Apollo solution for 0.5 h, and then stained with 4,6-diamidino-2-phenylindole (DAPI) for 0.5 h. Following that, the total number of blue fluorescently labeled cells and the number of red fluorescently labeled proliferating cells were observed and photographed under a confocal laser scanning microscope (Leica Microsystems, Germany). Cell proliferation rate = number of red fluorescent cells / number of blue fluorescent cells×100%.

### Transwell assay

Transwell system (pore size 8 μm; Corning Costar, USA) was used to measure cell migration. Invasion was detected using Transwell system with Matrigel (Millipore, USA). Forty-eight hours after transfection, cells suspended in serum-free medium were seeded into the upper chamber. The lower chamber contained medium supplemented with 20% FBS, which acted as a chemotactic agent. After 24 h of culture, cells that had migrated and invaded were fixed in methanol and stained with 0.1% crystal violet. IX71 inverted microscope (Olympus, Japan) was used to take the photographs.

### Dual-luciferase reporter assay

DLGAP1-AS1 sequence containing the predicted binding site for miR-515-5p was cloned into the pmirGLO dual-luciferase reporter gene expression vector (Promega, USA) to construct a reporter plasmid named pmirGLO-DLGAP1-AS1-WT (DLGAP1-AS1-WT), and mutated DLGAP1-AS1 sequence was used to construct pmirGLO-DLGAP1-AS1-Mut (DLGAP1-AS1-Mut). HEK293T cells were seeded onto a 96-well plate, and the above vectors were co-transfected with miR-515-5p mimic or miR-NC into the cells using Lipofectamine^TM^ 2000 (Invitrogen) following manufacturer instructions. Forty-eight hours after transfection, the luciferase activity was evaluated using the luciferase detection kit (Promega) following manufacturer instructions. Similarly, the targeting relationship between miR-515-5p and the 3′UTR of MARK4 was investigated.

### RNA immunoprecipitation (RIP) assay

RIP assay was conducted using the EZ-Magna-RIP kit (EMD Millipore). GC cells were lysed in RIP lysis buffer containing protease inhibitor cocktail (Roche Diagnostics, China). The lysate was then incubated with washing buffer and the RIP buffer containing magnetic beads conjugated with anti-argonaute 2 (Ago2) antibody or mouse IgG was added. Subsequently, immunoprecipitation complex was incubated with proteinase K and co-immunoprecipitated RNA was extracted with TRIzol method. Finally, qRT-PCR was performed.

### Western blot

Total protein was extracted from cells using RIPA buffer (Beyotime) supplemented with protease inhibitor. Proteins were separated by 10% SDS-PAGE and transferred to PVDF membranes (Millipore). After blocking with 5% skimmed milk at room temperature for 1 h, the membrane was rinsed with TBST 3 times for 10 min each time, and then incubated with primary antibodies anti-MARK4 (Abcam, China; ab5262; 1:1000) and anti-GAPDH (Abcam; ab8245; 1:1000) at 4°C overnight. After washing the membrane with TBST, the membrane was incubated with horseradish peroxidase (HRP)-labeled secondary antibody (Abcam; ab205718, 1:2000) at room temperature for 1 h. The membrane was then washed with TBST for 3 times. Ultimately, the ECL kit (Millipore) was used for the development of the protein bands and the gray value of each band was analyzed using ImageJ software (NIH, USA).

### Statistical analysis

All statistical analyses were performed using SPSS 23.0 (IBM, USA) and graphing was performed using GraphPad Prism 8 (GraphPad Prism, Inc., USA). Each experiment was repeated at least three times and the data are reported as means±SD. The Student's *t*-test or one-way analysis of variance was applied to compare the average of two or more groups. The correlation between DLGAP1-AS1 expression and clinicopathological variables was calculated by chi-squared test. P<0.05 indicated statistical significance.

## Results

### DLGAP1-AS1 expression was up-regulated in GC tissues and cells and related to adverse pathological characteristics

**Figure 1:**
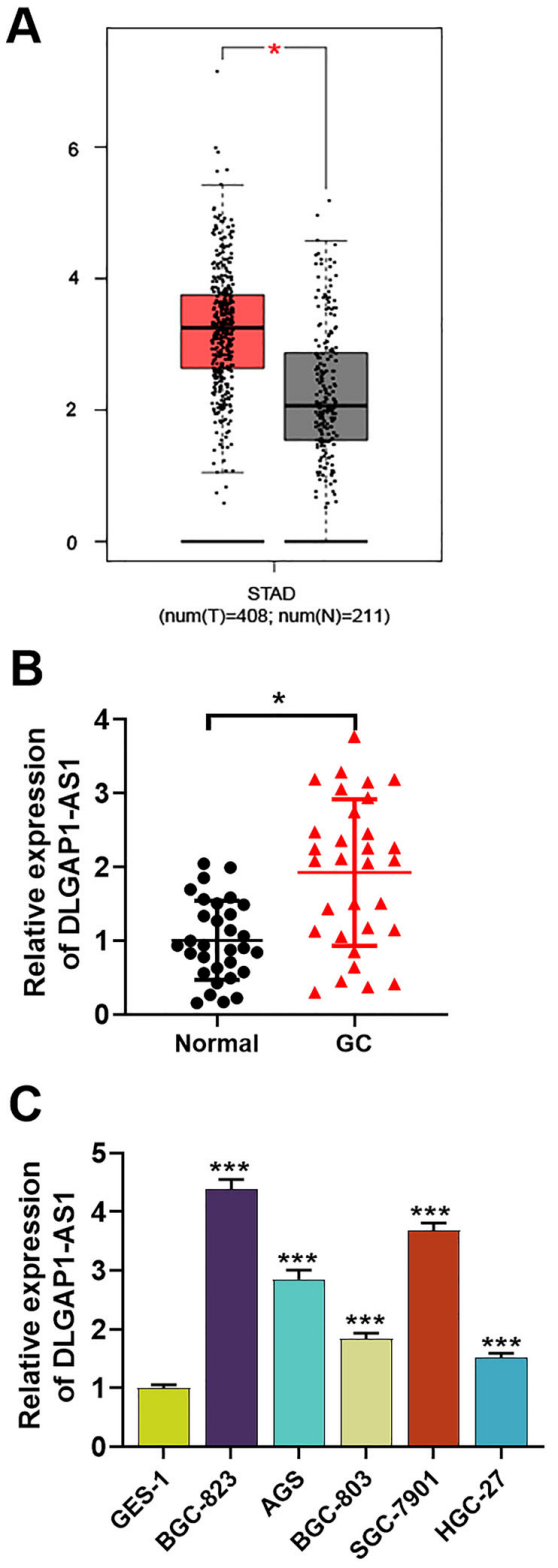
DLGAP1-AS1 is up-regulated in gastric cancer (GC) tissues and cells. **A**, Differential expression of DLGAP1-AS1 in 408 GC tissues and 211 non-cancer tissues in the GEPIA database (data are reported as median and interquartile range). **B**, qRT-PCR detected the relative expression of DLGAP1-AS1 in GC tissue (n=31) compared with matched normal gastric tissues. **C**, DLGAP1-AS1 expression levels in five gastric cancer cell lines (BGC-823, AGS, BGC-803, SGC-7901, and HGC-27) and normal gastric mucosal epithelial cell lines (GES-1) were detected by qRT-PCR. Data are reported as means±SD. *P<0.05, ***P<0.001 (Student's *t*-test or ANOVA).

By analyzing the Gene Expression Profiling Interactive Analysis (GEPIA) database (http://gepia.cancer-pku.cn/) (12), which is a web server for cancer and normal gene expression profiling and interactive analyses, it was found that DLGAP1-AS1 expression in GC tissues was significantly higher than that in normal tissues (Figure 1A). Additionally, qRT-PCR was carried out to examine DLGAP1-AS1 expression in GC tissues and matched normal gastric tissues of 31 patients, and, consistently, the data revealed that DLGAP1-AS1 was significantly up-regulated in GC tissues (Figure 1B). Furthermore, compared with human normal gastric mucosal epithelial cell line GES-1, DLGAP1-AS1 was highly expressed in five GC cell lines including BGC-823, AGS, BGC-803, SGC-7901, and HGC-27 (Figure 1C). Additionally, high DLGAP1-AS1 expression was associated with the enlargement of tumor size, higher TNM stage, and occurrence of lymph node metastasis (Table 2). These results implied that DLGAP1-AS1 may be essential for the tumor development and metastasis of GC.

**Table 2:** Association between DLGAP1-AS1 expressions and clinicopathologic features of patients with gastric cancer.

Clinicopathologic features	Cases n=31	DLGAP1-AS1 expression		P-value
		High 17	Low 14	
Age (years)				
<60	11	7	4	0.465
≥60	20	10	10	
Gender				
Male	15	6	9	0.108
Female	16	11	5	
Tumor size (cm)				
≤5	13	4	9	0.022*
>5	18	13	5	
Differentiation				
Poor	14	6	8	0.224
High/moderate	17	11	6	
TNM stage				
I	9	2	7	0.020*
II/III	22	15	7	
Lymph node metastasis				
Yes	21	15	6	0.007**
No	10	2	8	

The chi-squared test was used for statistical analyses. Asterisks indicate statistical significance.

### DLGAP1-AS **1 enhanced the proliferation, migration, and invasion of GC cells** *in vitro*

**Figure 2:**
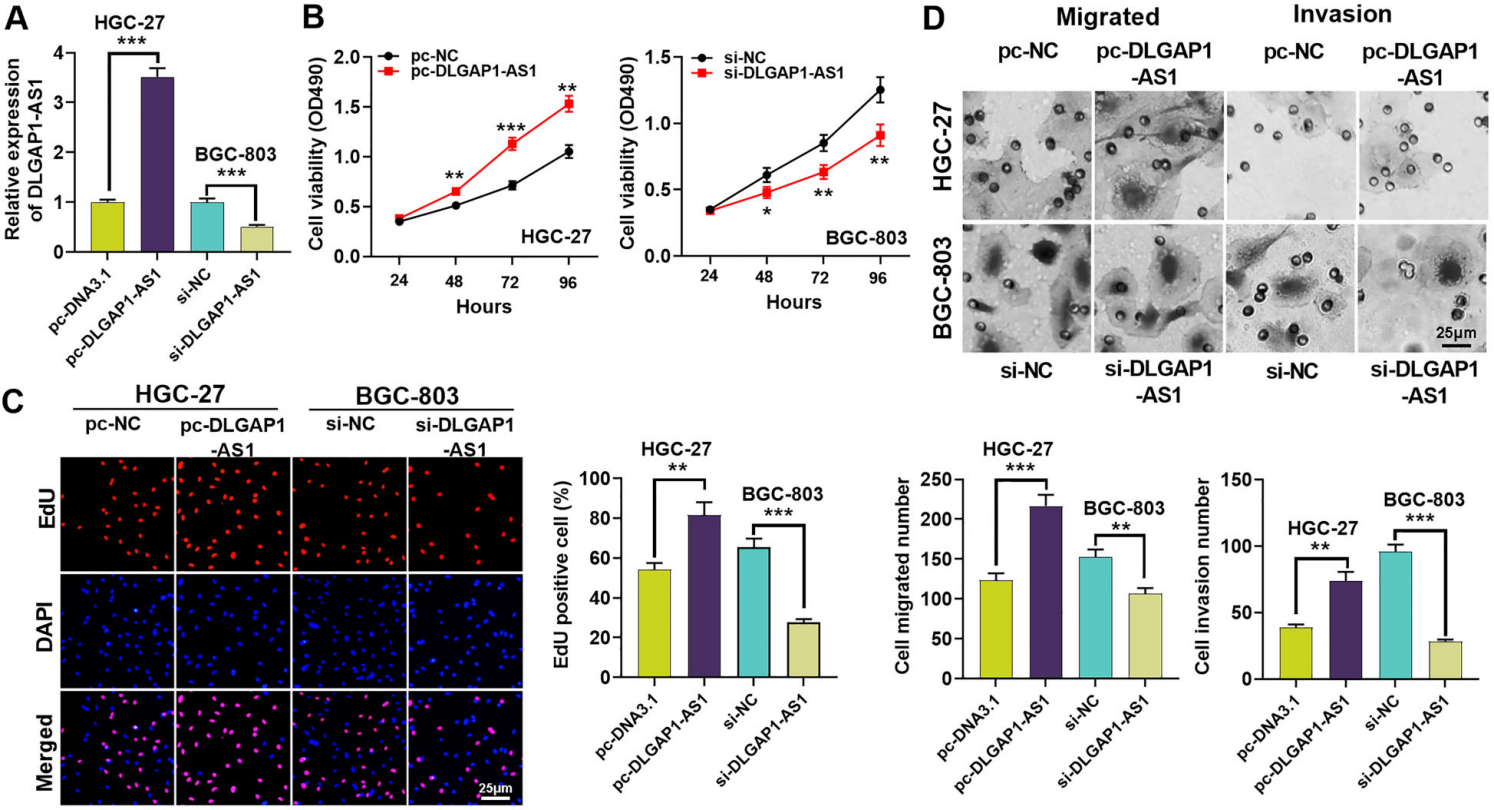
DLGAP1-AS1 can promote the proliferation, migration, and invasion of GC cells *in vitro.* **A**, BGC-803 cells were transfected with DLGAP1-AS1 siRNA (si-DLGAP1-AS1) and si-NC (negative control), and HGC-27 cells were treated with pc-DLGAP1-AS and pcDNA3.1 vectors. Then qRT-PCR was employed to analyze DLGAP1-AS1 expression. **B**, CCK-8 assay was performed to determine the viability of si-DLGAP1-AS1-transfected BGC-803 cells and pc-DLGAP1-AS1-transfected HGC-27 cells. **C**, EdU assay was used to determine the proliferation of si-DLGAP1-AS-transfected BGC-803 cells and pc-DLGAP1-AS1-transfected HGC-27 cells. **D**, Transwell assay was conducted to detect the migration and invasion of BGC-803 cells transfected with si-DLGAP1-AS1 and HGC-27 cells transfected with pc-DLGAP1-AS1. Data are reported as means±SD. *P<0.05, **P<0.01, and ***P<0.001 (ANOVA). **C** and **D**: scale bar 25 μm.

Next, we transfected pc-DLGAP1-AS1 into the HGC-27 cell line to highly express DLGAP1-AS1, the BGC-803 cell line was transfected with si-DLGAP1-AS1 to silence DLGAP1-AS1 expression, and the transfection efficiency was validated by qRT-PCR (Figure 2A). We then performed CCK-8 and EdU analyses and the results revealed that DLGAP1-AS1 overexpression enhanced cell viability and proliferation (Figure 2B and C). Furthermore, transwell experiments demonstrated that DLGAP1-AS1 overexpression facilitated the migration and invasion of HGC-27 cells, while DLGAP1-AS1 knockdown repressed these capabilities of BGC-803 cells (Figure 2D).

### DLGAP1-AS1 acted as a molecular sponge to directly interact with miR-515-5p

**Figure 3:**
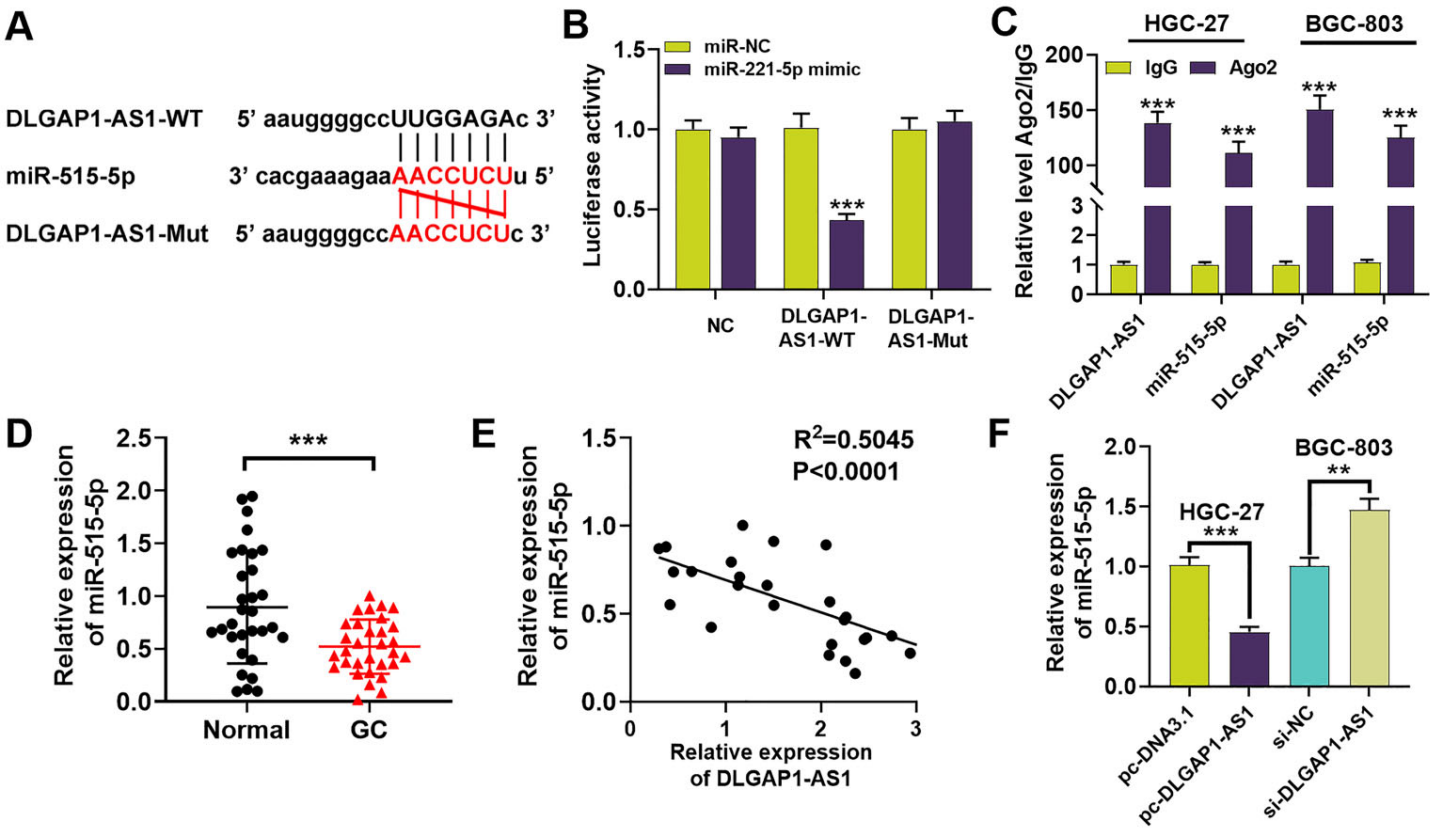
DLGAP1-AS1 directly interacts with miR-515-5p. **A**, Targeted binding sites wild type (WT) and mutant (Mut) between miR-515-5p and DLGAP1-AS1. **B**, Dual-luciferase reporter assay was used to validate the complementary base pairing between miR-515-5p and the 3'UTR of DLGAP1-AS1. **C**, The binding relationship between DLGAP1-AS1 and miR-515-5p was verified by RIP analysis. **D**, qRT-PCR detected miR-515-5p expression levels in 31 gastric cancer (GC) tissues and matched normal gastric tissues. **E**, The expression correlation between DLGAP1-AS1 and miR-515-5p in GC tissues was determined. **F**, qRT-PCR was used to detect the expression of miR-515-5p in pc-DLGAP1-AS1-transfected HGC-27 cells and si-DLGAP1-AS1-transfected BGC-803 cells. Data are reported as means±SD. **P<0.01 and ***P<0.001 (*t*-test or ANOVA).

According to the bioinformatics analysis with StarBase v2.0 (http://starbase.sysu.edu.cn/index.php) (13), we observed that DLGAP1-AS1 could potentially bind with miR-515-5p (Figure 3A). Dual-luciferase reporter gene assay revealed that miR-515-5p mimic markedly reduced the luciferase activity of DLGAP1-AS1-WT but did not affect the activity of DLGAP1-AS1-Mut (Figure 3B). Additionally, RIP assay further validated the interaction between miR-515-5p and DLGAP1-AS1 (Figure 3C). Subsequently, we found that miR-515-5p had a significantly increased expression in GC tissue compared to normal gastric epithelial tissue (Figure 3D). Pearson's correlation analysis indicated that DLGAP1-AS1 and miR-515-5p were negatively correlated in GC tissues (Figure 3E). Additionally, miR-515-5p expression was remarkably suppressed in pc-DLGAP1-AS-transfected HGC-27 cells, while miR-515-5p expression was up-regulated in si-DLGAP1-AS1-transfected BGC-803 cells (Figure 3F). These data suggested that DLGAP1-AS1 directly interacted with miR-515-5p and repressed its expression.

### miR-515-5p partially rescued the DLGAP1-AS1-induced tumor-promoting effects on GC cells

**Figure 4:**
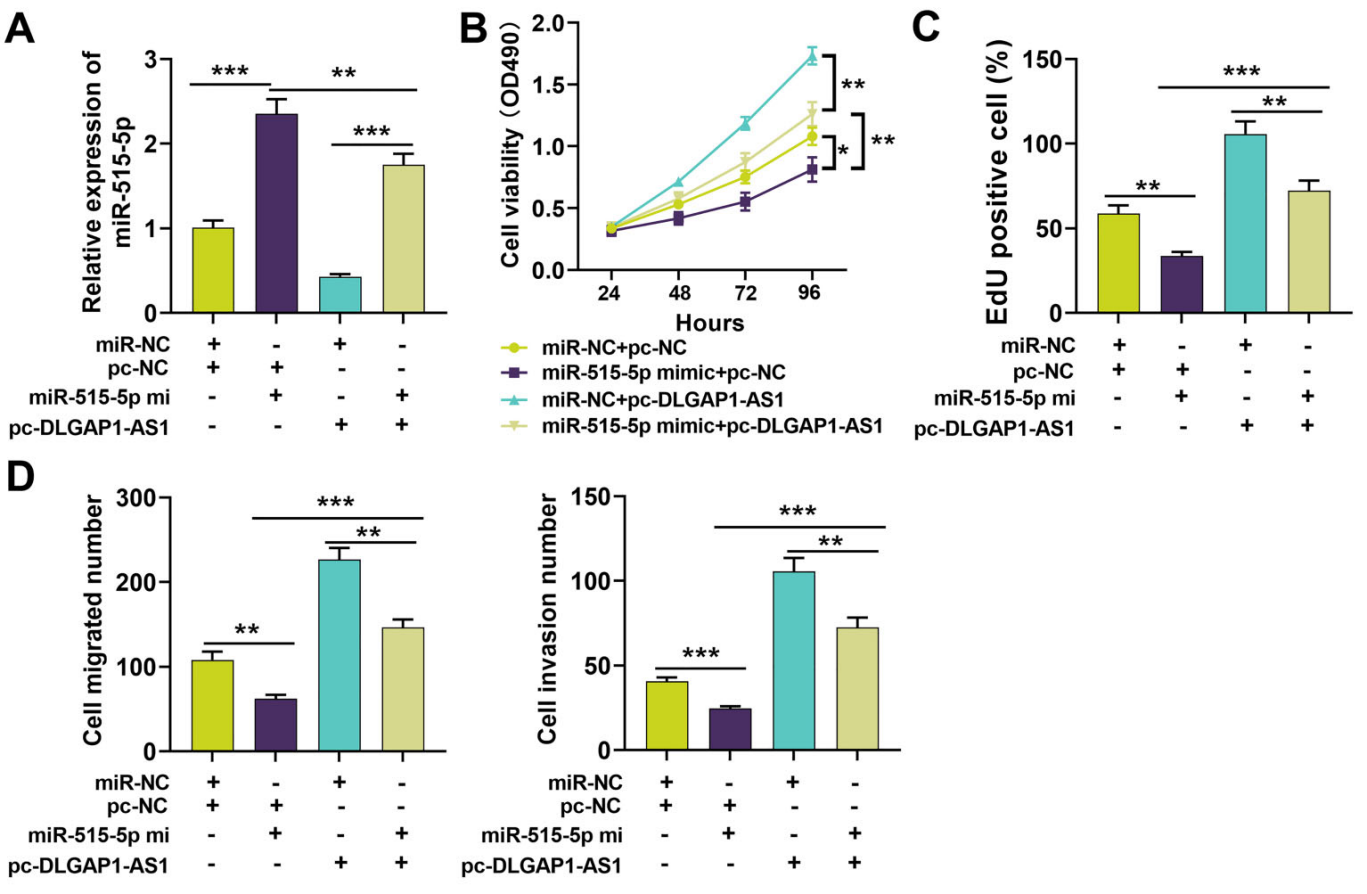
miR-515-5p partially rescued the DLGAP1-AS1-induced tumor-promoting effects on gastric cancer cells. **A**, miR-515-5p mimic and miR-NC (negative control) were transfected into the pc-DLGAP1-AS1-transfected HGC-27 cell line, and qRT-PCR was used to detect the expression of miR-515-5p in each group. **B**, CCK-8 was employed to detect the viability of cells in each group. **C**, EdU assay was used to determine the proliferation of cells in each group. **D**, Transwell assay was applied to detect the migration and invasion of cells in each group. Data are reported as means±SD. *P<0.05, **P<0.01, and ***P<0.001 (ANOVA).

To further confirm the role of DLGAP1-AS1/miR-515-5p axis in regulating the malignant biological behaviors of GC cells, we transfected miR-515-5p mimic into HGC-27 cells with DLGAP1-AS1 overexpression (Figure 4A). CCK-8 assay, EdU assay, and transwell assay revealed that, compared with the pc-DLGAP1-AS1 + miR-NC group, miR-515-5p could partially reverse cell proliferation, migration, and invasion promoted by DLGAP1-AS1 overexpression (Figure 4B-D). Overall, the findings implied that the cancer-promoting effects of DLGAP1-AS1 could probably be mediated by the inhibition of miR-515-5p.

### DLGAP1-AS1 regulated MARK4 expression by targeting miR-515-5p

**Figure 5:**
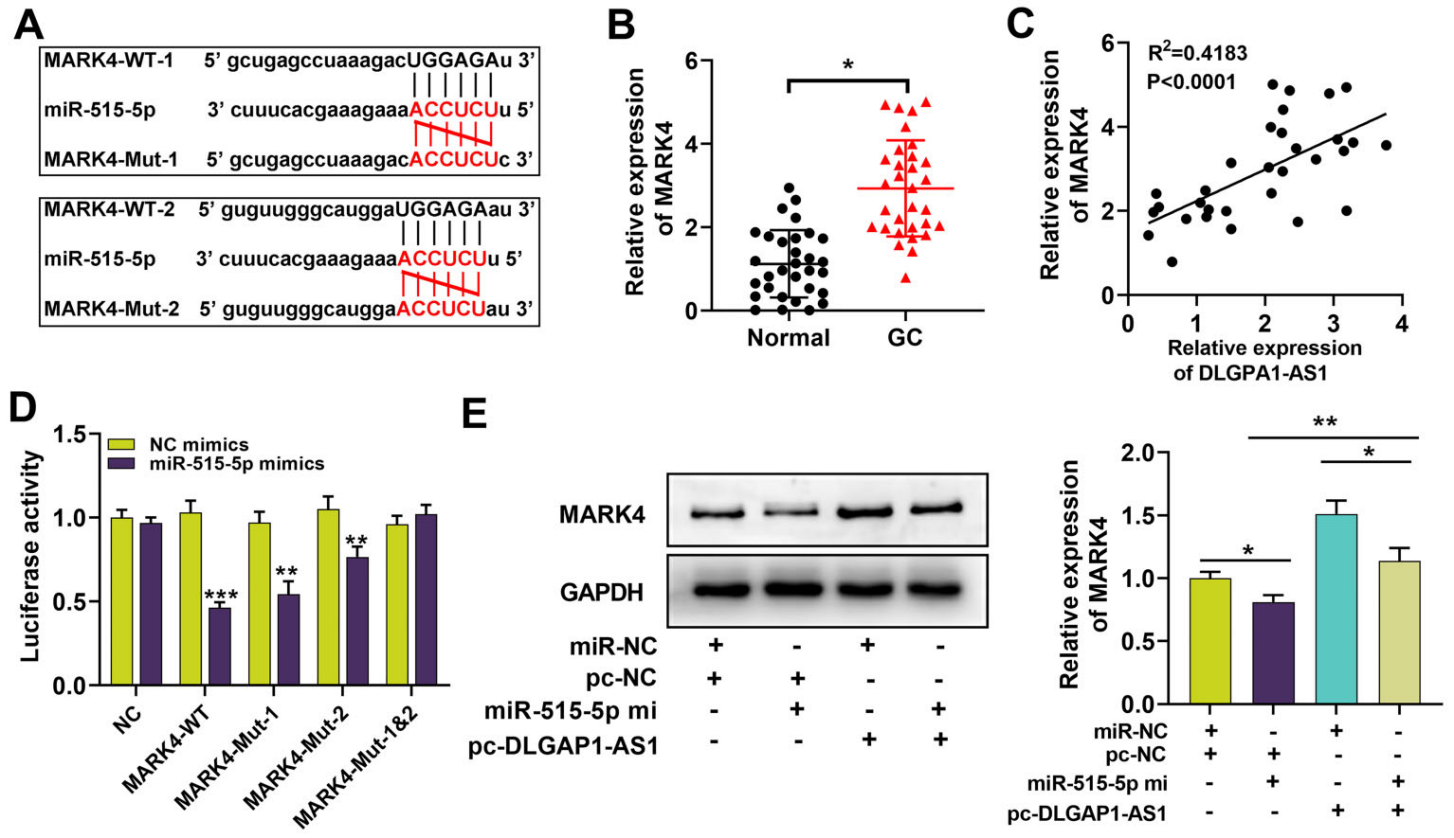
DLGAP1-AS1 regulates MARK4 expression by targeting miR-515-5p. **A**, Two targeted binding sites between miR-515-5p and MARK4 3' UTR. **B**, qRT-PCR was used to detect the expression of MARK4 in 31 gastric cancer (GC) patient tissues and matched normal gastric tissues. **C**, The expression correlation between MARK4 and miR-515-5p in GC tissues was determined. **D**, Dual-luciferase reporter assay was used to validate the complementary base pairing between miR-515-5p and the 3′UTR of MARK4. **E**, MARK4 expression in HGC-27 cell line was detected by western blot after transfection. Data are reported as means±SD. *P<0.05, **P<0.01, and ***P<0.001 (ANOVA).

It is reported that miR-515-5p directly targets MARK4 3′ UTR, represses its expression, and participates in regulating the biological behaviors of cancer cells (11). Consistently, bioinformatics analysis with TargetScan database predicted 2 binding sites between miR-515-5p and the 3′UTR of MARK4 (Figure 5A). qRT-PCR showed that MARK4 was markedly up-regulated in GC tissues compared with matched normal gastric tissues (Figure 5B). Pearson's correlation analysis indicated a negative correlation between MARK4 expression and miR-515-5p expression in GC samples (Figure 5C). Additionally, dual-luciferase reporter gene experiments showed that miR-515-5p mimic markedly suppressed the luciferase activity of MARK4-WT, MARK4-Mut-1, MARK4-Mut-2 reporters, but had no significant effect on the luciferase activity of MARK4-Mut-1 and -2 reporter, which validated the 2 binding sites between miR-515-5p and MARK4 3′UTR (Figure 5D). Furthermore, by western blot, it was revealed that DLGAP1-AS1 overexpression enhanced MARK4 expression in HGC-27 cells, while transfection of miR-515-5p mimic attenuated this effect (Figure 5E). Additionally, through Kaplan-Meier analysis (http://www.kmplot.com/), it was found that MARK4 high expression was closely associated with unfavorable prognosis in GC patients: patients with high expression of MARK4 had faster first progression (FP), shorter overall survival (OS) time, and post-progression survival (PPS) time (Supplementary Figure S1).

## Discussion

Every year, about 700,000 persons die from GC worldwide (14). Exploring the mechanism of GC progression is still in needed urgently. In recent years, accumulating lncRNAs have been confirmed to be involved in carcinogenesis and development of GC (15). For instance, lncRNA AK023391 exerts its cancer-promoting effect by activating the PI3K/Akt signaling pathway (16); SNHG8 enhances the proliferation and invasion of GC cells by targeting the miR-491/PDGFRA axis (17). Recent studies suggest that DLGAP1-AS1 promotes the progression of hepatocellular carcinoma by regulating miR-26a/b-5p and miR-486-5p (7,18). In this research, we discovered that DLGAP1-AS1 was highly expressed in GC tissues, and its high expression was closely related to tumor size, TNM stage, and lymph node metastasis. Functional experiments revealed that DLGAP1-AS1 could facilitate the proliferation, migration, and invasion of GC cells, suggesting that DLGAP1-AS1 played a pro-cancer role in GC. The present work proved that DLGAP1-AS1 was an oncogenic lncRNA in GC, which indicated it could be a promising biomarker and therapy target. Interestingly, our results are in agreement with a recent study that reported that the increased expression of DLGAP1-AS1 in GC tissues correlates with tumor size, TNM stage, lymph node metastasis, distant metastasis, and poor prognosis of the patients (19).

lncRNA acts as a competitive endogenous RNA (ceRNA), so-called molecular sponge, to interact with miRNA and inhibit miRNA expression to reduce the miRNA repressive effect on targeting mRNA (20). This mechanism is involved in regulating the malignant biological behavior of cancer cells. For instance, lncRNA AGAP2-AS1, as a ceRNA, exerts a pro-cancer effect in liver cancer by up-regulating ANXA11 expression via sponging miR-16-5p (21). It has been reported that lncRNA GAS5 regulates the Hippo signaling pathway through repressing miR-181c-5p and antagonizes the chemoresistance of pancreatic cancer cells (22). In GC, lncRNA HOTAIR suppresses miR-331-3p and up-regulates human epithelial growth factor receptor 2 (Her2) expression, thereby playing a carcinogenic role in the pathogenesis of GC (23). LINC01133 up-regulates APC expression and activates Wnt/β-catenin pathway by serving as a ceRNA for miR-106a-3p (24). In the present work, we demonstrated that DLGAP1-AS1 contained a targeted binding site for miR-515-5p and DLGAP1-AS1 could negatively regulate the expression of miR-515-5p. Importantly, miR-515-5p reversed the oncogenic function of DLGAP1-AS. These results suggested that DLGAP1-AS1 functioned as a molecular sponge for miR-515-5p in GC.

Previous research has revealed that miR-515-5p participates in the progression of a variety of cancers including prostate cancer, breast cancer, non-small cell lung cancer, colorectal cancer, and GC (25–33). For example, the expression level of miR-515-5p is significantly associated to overall survival time of rectal cancer patients (31). miR-515-5p is repressed by estrogen receptor alpha and it plays a tumor-suppressive role in breast cancer (32). Moreover, miR-515-5p also has great importance in GC progression (28 [Bibr B29] [Bibr B30],^33^). It targets X chromosome-linked inhibitor of apoptosis (XIAP) to repress the malignant phenotypes of GC cells (33), and LINC00511 enhances the proliferation and invasion of GC cells by inhibiting miR-515-5p (28). In the present work, it was demonstrated that miR-515-5p exerted its tumor-suppressive function in GC by inhibiting MARK4, and this regulatory relationship between miR-515-5p and MARK4 was consistent with previous reports (11,26 [Bibr B27]).

MARK4 is a Ser/Thr kinase. Previous research has shown that MARK4 is up-regulated in gliomas, hepatocellular carcinoma, prostate cancer, and others (8-10,34–37). In the present work, it was demonstrated that DLGAP1-AS1/miR-515-5p axis modulated the expression of MARK4. Importantly, bioinformatics analysis indicated that the overexpression of MARK4 in GC tissues was correlated with unfavorable prognosis. Collectively, our results indicated that DLGAP1-AS1 regulated MARK4 expression in a miR-515-5p-dependent manner to promote GC progression. MARK4 is a crucial modulator of multiple signaling pathways. For example, MARK4 expression inhibited Hippo signaling and facilitated breast cancer cell proliferation and metastasis (10,26). These studies hint that DLGAP1-AS1 can probably regulate cancer-related signal pathways such as Hippo to promote GC progression, which needs to be explored and validated in future studies.

**Figure 6:**
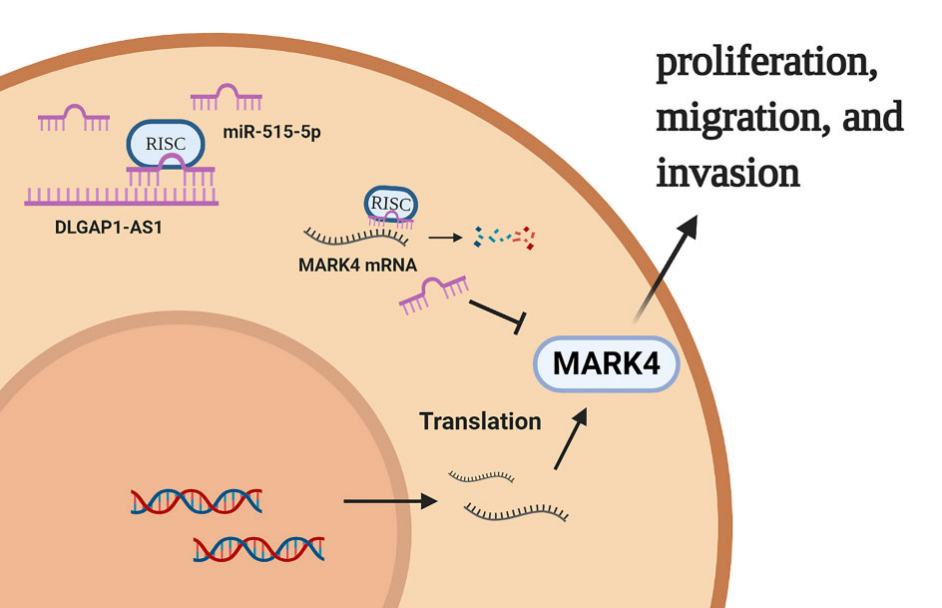
The underlying mechanism of how DLGAP1-AS1 is involved in the progression of gastric cancer.

In summary, this research implies that DLGAP1-AS1 was up-regulated in GC tissues and was closely associated with tumor size, TNM stage, and lymph node metastasis. DLGAP1-AS1 mediated the proliferation, migration, and invasion of GC cells by modulating the miR-515-5p/MARK4 axis (Figure 6). Our data indicated that DLGAP1-AS1 is a promising biomarker and target for GC diagnosis and treatment. The data used to support the findings of this study are available from the corresponding author upon request.

## Supplementary Material

Click here to view [pdf].
